# Publication characteristics of oral presentations at the European Society of Cardiovascular Radiology congresses

**DOI:** 10.3389/fcvm.2026.1890083

**Published:** 2026-08-25

**Authors:** Hamza Eren Güzel, Ali Salbas, Özgür Yaşar, Alperen Erboğa, Babak Saravi

**Affiliations:** 1 Department of Radiology, İzmir City Hospital, University of Health Sciences, İzmir, Türkiye; 2 Department of Radiology, İzmir Katip Çelebi University Atatürk Training and Research Hospital, İzmir, Türkiye; 3 Department of Oral, Maxillofacial and Facial Plastic Surgery, Medical Faculty and University Hospital Düsseldorf, Heinrich-Heine-University Düsseldorf, Düsseldorf, Germany

**Keywords:** bibliometric analysis, cardiovascular imaging, congress abstracts, European Society of Cardiovascular Radiology, oral presentations, publication rate, radiology research, scientific dissemination

## Abstract

**Background:**

Scientific congresses are important platforms for the early dissemination of research findings; however, not all presented abstracts subsequently reach full-text publication. Although congress-to-publication outcomes have been examined for several radiology meetings, the European Society of Cardiovascular Radiology (ESCR) congress has not been specifically evaluated. This study aimed to evaluate the publication outcomes of ESCR oral presentations, explore univariable associations between presentation characteristics and subsequent publication, and characterize temporal changes in research patterns.

**Methods:**

Oral presentations from the ESCR congresses held between 2019 and 2022 were retrospectively reviewed. The 2018 and 2023 joint meetings with the European Society of Thoracic Imaging were excluded to reduce heterogeneity. Abstracts published before the corresponding congress and duplicate presentations were excluded. Publication was defined as subsequent full-text original research indexed in PubMed; searches were conducted between April 24 and May 10, 2026. Country of origin, subspecialty, study design, and collaboration type were recorded. Associations with publication outcome were assessed using chi-square tests or an exact test for sparse country-level data. A standardized 36-month analysis was used for congress-year comparisons, and temporal changes in study design and collaboration were evaluated.

**Results:**

Of 291 oral presentations, 145 were subsequently published as PubMed-indexed full-text original research articles, yielding a publication rate of 49.8%. Median time to publication was 13.0 months, and 89.7% of published studies appeared within three years. Publication outcome differed significantly by country of origin, subspecialty, and study design, whereas collaboration type was not significantly associated with publication. Publication rates did not differ across congress years (*p* = 0.726), including with standardized 36-month follow-up (*p* = 0.478), whereas study-design and collaboration distributions changed significantly over time (both *p* < 0.001).

**Conclusion:**

Approximately half of ESCR oral presentations progressed to PubMed-indexed full-text original research, and most identified publications appeared within three years. Publication conversion remained stable across congress years, whereas the design and collaborative structure of presented research changed over time. As the first ESCR-specific congress-to-publication analysis, these findings provide a benchmark for research dissemination in cardiovascular radiology; however, restriction to PubMed may underestimate the overall full-text publication rate.

## 1 Introduction

Scientific congresses play an important role in the dissemination of new research findings, academic networking, and the development of collaborative research environments. Oral presentations delivered at these meetings often represent preliminary or ongoing studies and provide researchers with an opportunity to receive feedback before submission to peer-reviewed journals. However, not all presented abstracts subsequently achieve full-text publication. Accordingly, the proportion of congress presentations that subsequently reach full-text publication is commonly used as an indirect indicator of research dissemination and meeting-related scientific output (1–4).

The European Society of Cardiovascular Radiology (ESCR) annual congress is an international scientific meeting focused on cardiovascular CT, cardiovascular MRI, vascular imaging, and related imaging technologies. Cardiovascular imaging is a rapidly evolving and technology-intensive subspecialty, with continuing developments in CT, MRI, quantitative imaging, and image-based clinical research (5, 6). In this setting, evaluating congress output can provide information not only on whether presented work reaches peer-reviewed publication but also on how the design and collaborative structure of research change over time.

Previous studies have evaluated the full-text publication outcomes of congress abstracts across several medical specialties (1–4). Within radiology, publication rates and publication-related characteristics have been examined for general radiology and subspecialty meetings, including ECR, RSNA, ESGAR, ESNR, interventional radiology, and national radiology congresses (7–17). Despite this established literature, to the best of our knowledge, no previous study has specifically evaluated publication outcomes from the ESCR congress. This represents a relevant gap because ESCR provides a focused view of research dissemination within cardiovascular imaging. Evaluating publication rate and timing together with temporal changes in study design and collaboration patterns may therefore help characterize the evolution of scientific activity in this subspecialty rather than providing an isolated publication percentage alone. Accordingly, the present study aimed to determine the publication rate and time to publication of ESCR oral presentations, explore univariable associations between presentation characteristics and subsequent publication, and examine changes in research characteristics across congress years.

## 2 Materials and methods

As the study did not involve patient data or human participants, ethical approval and informed consent were not required. This study evaluated the publication outcomes of oral presentations delivered at the annual congresses of the ESCR. Abstracts presented between 2019 and 2022 were included in the analysis. The 2018 and 2023 congresses were excluded because these meetings were jointly organized with the European Society of Thoracic Imaging (ESTI), potentially introducing heterogeneity in scientific content and congress structure. Previous studies have shown that most congress abstracts that ultimately achieve full-text publication are published within the first three years after presentation (1, 10, 12, 14, 16). Therefore, more recent congresses, including the 2024 and 2025 meetings, were not included to ensure an adequate follow-up period for potential full-text publication. Abstracts were obtained from the official ESCR abstract books published in the International Journal of Cardiovascular Imaging (18–21).

Only oral presentations were included in the analysis, as the congress abstract books did not contain poster presentations. Initially, a total of 316 oral presentations were identified. Abstracts that had already been published as full-text articles before the corresponding congress date (*n* = 22) were excluded. In addition, three duplicate abstracts that had been presented at more than one congress meeting were identified and excluded from the final analysis. The final study population consisted of 291 oral presentations.

For each abstract, the following variables were recorded: country of the first author, subspecialty category, study design, collaboration type, presentation type, and publication status. For descriptive purposes, presentation type was classified as original research, review, case report, meta-analysis, or systematic review according to the content of the congress abstract. Subspecialty categories were predefined by the study team according to the predominant cardiovascular imaging topic of each abstract and were classified as myocardial, coronary, valvular, vascular, congenital, aortic, pulmonary, or other imaging. The ‘other’ subspecialty category included presentations that could not be assigned to one of the seven predefined cardiovascular subspecialty groups. Study design was categorized as retrospective, prospective, or other. The ‘other’ study-design category comprised review-type presentations, including reviews, systematic reviews, and meta-analyses (*n* = 42); case-based presentations (*n* = 6); and five original-research presentations that could not be classified as retrospective or prospective and were predominantly survey-, phantom-, or technical-type studies. This heterogeneous category was retained because these presentations did not meet the predefined criteria for either retrospective or prospective study design. Collaboration type was included because institutional and international collaboration has been evaluated as a potential correlate of subsequent publication in previous congress-outcome studies (10, 12, 17). Collaboration was classified as local when all authors were affiliated with the same institution, national when authors were affiliated with more than one institution within the same country, and international when at least one author was affiliated with an institution in a different country. Thus, the presence of a single co-author with a foreign institutional affiliation was sufficient for classification as international collaboration.

Publication status was determined through a systematic search of PubMed. PubMed was selected because the primary outcome was defined *a priori* as publication as a full-text original research article indexed in PubMed and because this approach facilitates comparison with previous radiology congress-outcome studies (7–17). Web of Science and Scopus were not searched; consequently, articles indexed exclusively in other databases may not have been identified. Searches were conducted between April 24, 2026, and May 10, 2026. Only full-text original research articles were considered published studies; case reports, reviews, letters, and editorials were excluded. Four radiologists performed the searches in two independent reviewer pairs. Each abstract-publication match was independently evaluated by two radiologists within the assigned pair. Searches were initially conducted using the surname and initials of the first author; when no corresponding publication was identified, additional searches used co-author names and keywords derived from the abstract title. A potential publication was accepted as corresponding to the congress abstract when the research topic and principal methodology were concordant and at least one author was shared between the abstract and full-text article. Changes in title wording, author order, or sample size were permitted when the article clearly represented the same study or an expanded version of the abstract. Substantial differences in study population, primary objective, or methodology were considered evidence against a match. Disagreements were resolved by reviewer consensus, with unresolved cases adjudicated after discussion with the lead radiologist and first author (H.E.G.).

For published studies, the journal name and publication date were recorded. When articles were available online ahead of print or as early online publications, the first online publication date was used for analysis. Publication time was calculated in days from the start date of the corresponding congress to the first online publication date. The congress start dates used for calculation were October 24, 2019; October 15, 2020; October 21, 2021; and October 27, 2022 (18–21). Publication time in months was calculated by dividing the number of elapsed days by 30.44.

### 2.1 Statistical analysis

All statistical analyses were performed using IBM SPSS Statistics (version 27, IBM Corp., Armonk, NY, USA). Categorical variables were presented as number and percentage. The distribution of publication time was assessed using the Shapiro–Wilk test together with visual inspection of its distribution. Because publication time was non-normally distributed (Shapiro–Wilk *p* < 0.001), it was primarily summarized as median with interquartile range (IQR) and range; mean and standard deviation (SD) were provided as additional descriptive measures. A two-sided *p*-value of <0.05 was considered statistically significant.

Associations between publication outcome and congress year, subspecialty category, collaboration type, and study design were evaluated using Pearson's chi-square test. Following the significant overall study-design test, *post hoc* pairwise comparisons were performed only among the three study-design categories using 2 × 2 Pearson chi-square tests. Because three pairwise comparisons were performed, Bonferroni-adjusted *p*-values were calculated by multiplying each unadjusted *p*-value by three. To account for the different total follow-up durations available for the four congress years, publication rates were additionally compared within a standardized 36-month follow-up window after each congress. Temporal changes in the distributions of study design and collaboration type across congress years were also evaluated using Pearson's chi-square tests.

Because country-of-origin data contained multiple sparse and zero cells, the association between country and publication outcome was evaluated across all 32 countries using the Fisher-Freeman-Halton exact test with Monte Carlo estimation rather than conventional logistic regression. Countries with fewer than eight oral presentations were combined under the ‘Other’ category only for descriptive presentation in the main table; this threshold was used to avoid displaying numerous very small country groups individually and did not affect inferential testing. The complete distribution for all 32 countries is provided in Supplementary Table S1. No country-specific logistic regression model was fitted because sparse groups and complete separation in several countries would yield unstable conventional estimates.

## 3 Results

**Figure 1:**
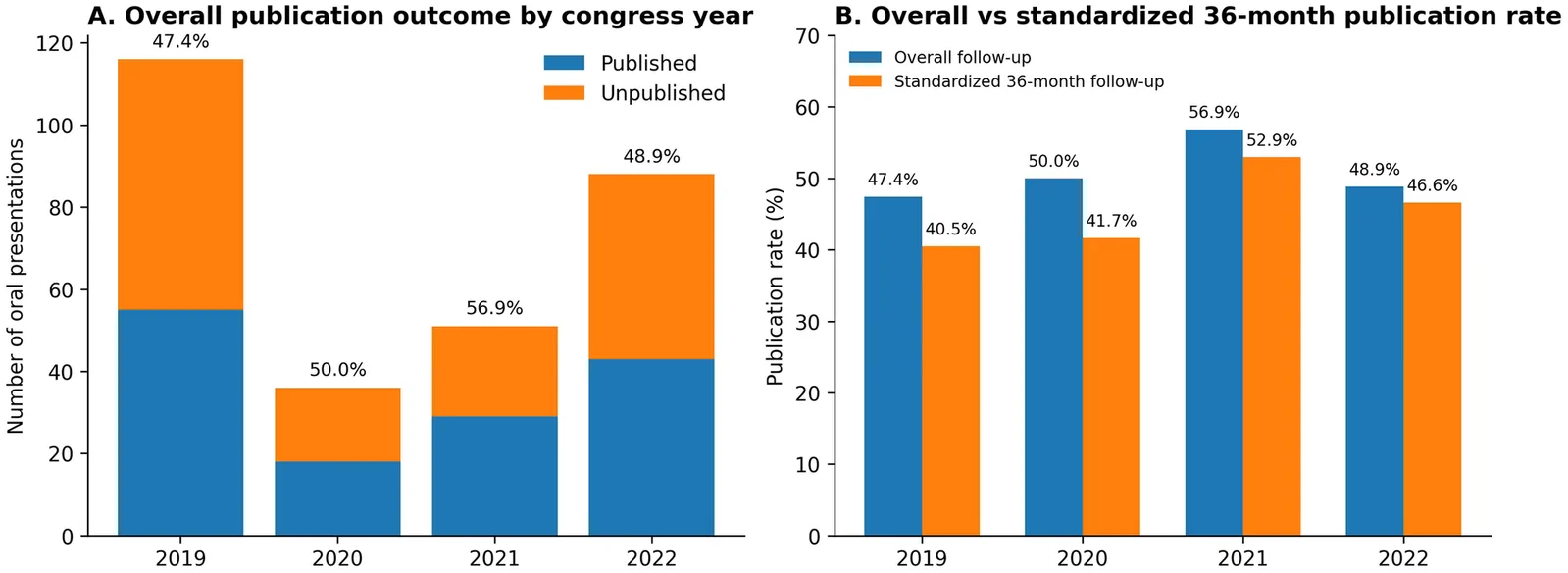
Temporal publication outcomes of ESCR oral presentations. **(A)** Number of oral presentations by congress year, stratified according to subsequent publication status; percentages above the bars indicate the overall publication rate for each congress year. **(B)** Overall publication rates and publication rates within a standardized 36-month follow-up period. No significant difference was observed across congress years for either overall publication rates (*p* = 0.726) or standardized 36-month publication rates (*p* = 0.478).

A total of 291 oral presentations from the ESCR congresses held between 2019 and 2022 were included in the final analysis. At the time of congress presentation, 243 (83.5%) were original research presentations, 36 (12.4%) were reviews, 6 (2.1%) were case reports, 4 (1.4%) were meta-analyses, and 2 (0.7%) were systematic reviews. Of all 291 presentations, 145 (49.8%) were subsequently published as full-text original research articles indexed in PubMed. The median time to publication was 13.0 months (IQR, 4.8–25.2 months; range, 0.1–75.5 months), with a mean of 16.7 months (SD, 14.1 months). Among the 145 published studies, 69 (47.6%) were published within the first year after presentation, whereas 130 (89.7%) were published within the first three years. The minimum interval of 0.1 months corresponded to an article published online three days after the start of the 2022 congress. The numbers of oral presentations were 116, 36, 51, and 88 in 2019, 2020, 2021, and 2022, respectively. Corresponding overall publication rates were 47.4% (55/116), 50.0% (18/36), 56.9% (29/51), and 48.9% (43/88), with no significant difference across congress years (*χ*^2^ = 1.31, *p* = 0.726; Figure 1A). When all congress years were restricted to the same 36-month follow-up interval, publication rates were 40.5% (47/116), 41.7% (15/36), 52.9% (27/51), and 46.6% (41/88), respectively, and again did not differ significantly across years (*χ*^2^ = 2.48, *p* = 0.478; Figure 1B).

**Figure 2:**
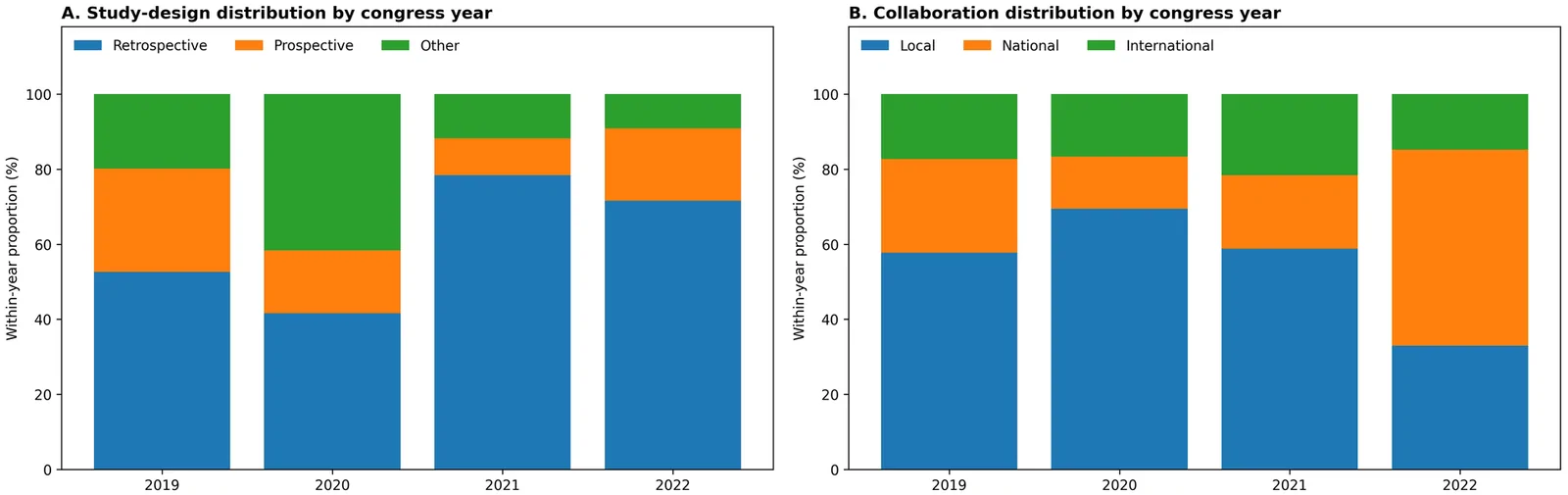
Temporal changes in the characteristics of ESCR oral presentations. **(A)** Distribution of retrospective, prospective, and other study designs by congress year. **(B)** Distribution of local, national, and international collaboration by congress year. Percentages represent within-year proportions. Both study-design and collaboration distributions changed significantly across congress years (*p* < 0.001 for each).

Research characteristics changed significantly across congress years (Figure 2). Retrospective studies accounted for 51.7%, 41.7%, 78.4%, and 71.6% of presentations in 2019, 2020, 2021, and 2022, respectively, with a significant difference in study-design distribution across years (*χ*^2^ = 30.39, *p* < 0.001; Figure 2A). Collaboration patterns also differed significantly over time (*χ*^2^ = 30.11, *p* < 0.001; Figure 2B). National collaborations represented 25.0%, 13.9%, 19.6%, and 52.3% of presentations in 2019, 2020, 2021, and 2022, respectively, whereas the proportion of local studies decreased to 33.0% in 2022.

The 145 published studies appeared across 55 different journals. European Radiology was the most common publication venue, accounting for 13.0% (19/145) of all published articles, followed by European Journal of Radiology and La radiologia medica, each representing 6.2% (9/145) of the publications (Table 1).

**Table 1:** Distribution of published articles according to journals.

Journal	*n*	%
*European Radiology*	19	13.0
*European Journal of Radiology*	9	6.2
*La radiologia medica*	9	6.2
*Journal of Clinical Medicine*	7	4.8
*Radiology: Cardiothoracic Imaging*	7	4.8
*Diagnostics*	5	3.5
*Insights into Imaging*	5	3.5
*Journal of Thoracic Imaging*	5	3.5
*Scientific Reports*	5	3.5
*The International Journal of Cardiovascular Imaging*	5	3.5
Other journals (< 5 publications)	69	47.5
Total	145	100

Journals with at least five publications are presented individually, whereas journals with fewer publications were grouped under the ‘Other journals' category. This category comprised 69 publications distributed across 45 different journals.

A total of 32 countries contributed oral presentations to the ESCR congresses between 2019 and 2022. Eleven countries had at least eight presentations and are displayed individually in Table 2, while the full country distribution is provided in Supplementary Table S1. Italy contributed the highest number of oral presentations (*n* = 92, 31.6%), followed by Germany (*n* = 47, 16.2%) and South Korea (*n* = 18, 6.2%). Publication outcome differed significantly according to country of origin (Fisher-Freeman-Halton exact test with Monte Carlo estimation, *p* < 0.001). Among countries with at least eight presentations, the highest observed publication rates were in Austria and the Netherlands (both 100.0%, 8/8), followed by Switzerland (75.0%, 6/8). These percentages should be interpreted cautiously because several country groups contained small numbers of presentations.

**Table 2:** Publication outcomes by country of origin.

Country	Presentations (n)	Published *n* (%)
Italy	92	53 (57.6%)
Germany	47	29 (61.7%)
South Korea	18	10 (55.6%)
Greece	15	1 (6.7%)
United Kingdom	10	6 (60.0%)
Romania	9	0 (0.0%)
Spain	9	1 (11.1%)
Austria	8	8 (100.0%)
France	8	5 (62.5%)
Netherlands	8	8 (100.0%)
Switzerland	8	6 (75.0%)
Other	59	18 (30.5%)

Countries with at least eight oral presentations are displayed individually in the main table for readability. Countries with fewer than eight presentations (*n* = 21) were combined under the ‘Other’ category for descriptive presentation only. The overall association between country of origin and publication outcome was evaluated across all 32 countries using the Fisher-Freeman-Halton exact test with Monte Carlo estimation (*p* < 0.001). The complete country-level distribution is provided in Supplementary Table S1.

The most frequently represented subspecialties were myocardial (*n* = 101, 34.7%) and coronary imaging (*n* = 75, 25.8%). Publication outcome differed significantly according to subspecialty category (*χ*^2^ = 20.75, *p* = 0.004). Among the subspecialties, pulmonary imaging demonstrated the highest publication rate (91.7%, 11/12), followed by valvular imaging (59.4%, 19/32). Detailed publication rates according to subspecialty are presented in Table 3.

**Table 3:** Publication rates across subspecialty categories, collaboration types, and study designs.

Characteristic	Presentations (n)	Published *n* (%)
Subspecialty		
Myocardial	101	55 (54.5%)
Coronary	75	34 (45.3%)
Valvular	32	19 (59.4%)
Vascular	20	8 (40.0%)
Other	18	10 (55.6%)
Congenital	18	4 (22.2%)
Aorta	15	4 (26.7%)
Pulmonary	12	11 (91.7%)
*p*	-	**0.004**
Collaboration		
Local	151	69 (45.7%)
National	90	49 (54.4%)
International	50	27 (54.0%)
*p*	-	0.342
Study design		
Retrospective	178	103 (57.5%)
Prospective	60	28 (46.7%)
Other	53	14 (26.9%)
*p*	-	**< 0.001**

Data are presented as number (percentage). Significant *p*-values are shown in bold. The ‘other’ subspecialty category includes presentations that did not fit into the seven predefined cardiovascular subspecialty groups. The ‘other’ study-design category comprised review-type presentations (*n* = 41), case-based presentations (*n* = 6), and five original-research presentations that could not be classified as retrospective or prospective. Bonferroni-adjusted pairwise comparisons for study design are reported in the Results section.

According to collaboration type, local collaboration was the most frequent category (*n* = 151, 51.9%), followed by national (*n* = 90, 30.9%) and international collaboration (*n* = 50, 17.2%) (Table 3). Publication rates did not differ significantly according to collaboration type (*χ*^2^ = 2.15, *p* = 0.342). National and international collaborations had publication rates of 54.4% and 54.0%, respectively, compared with 45.7% for local studies.

Retrospective studies represented the most common study-design category (*n* = 178, 61.2%). The highest publication rate was observed in retrospective studies (57.9%, 103/178), whereas the lowest rate was identified in the ‘other’ study-design category (26.4%, 14/53) (Table 3). Publication outcome differed significantly according to study design (*χ*^2^ = 16.46, *p* < 0.001). Following Bonferroni correction for three pairwise comparisons, retrospective studies had a significantly higher publication rate than studies classified as ‘other’ (adjusted *p* < 0.001). No significant difference was observed between retrospective and prospective studies (adjusted *p* = 0.395) or between prospective and ‘other’ studies (adjusted *p* = 0.079).

## 4 Discussion

In this cohort, 49.8% of ESCR oral presentations were subsequently identified as full-text original research articles indexed in PubMed, and most identified publications appeared within three years. To the best of our knowledge, this is the first congress-to-publication analysis focused specifically on ESCR. Importantly, publication conversion remained relatively stable across congress years, including after standardization to an equal 36-month follow-up period, whereas study-design and collaboration patterns changed substantially over time. The study therefore provides both a benchmark for dissemination of cardiovascular imaging research and a longitudinal view of how the structure of research presented at this subspecialty congress evolved during the study period. These findings should not be interpreted as direct measures of research quality or as evidence of causal determinants of publication.

The publication rate observed in the present study was higher than the pooled full-publication rate of 37.3% reported in a comprehensive systematic review of congress abstracts across medical specialties (1). In radiology, earlier studies reported publication rates of 37% for ASNR abstracts and 33% for RSNA neuroradiology abstracts presented in 1993, 33% for orally presented original studies at the 1995 RSNA Scientific Assembly, and 35.4% and 50.5% for RSNA and ECR abstracts presented by Korean investigators, respectively (13–15). An analysis of major interventional radiology meetings reported an overall publication rate of 44.9% (16). More recent European radiology data are closer to the present findings: 46.5% of ECR 2019 oral presentations were subsequently published, publication rates from ESGAR meetings ranged from 39.5% in 2000–2001 to more than half of oral presentations in 2019–2022, and 47.3% of ESNR oral presentations from 2018 to 2022 reached publication (9–12). Thus, the 49.8% rate observed in the present study is broadly comparable with contemporary European radiology subspecialty meetings. Differences across studies may reflect congress type, study period, abstract selection, and publication-search methodology rather than differences in research quality alone.

The median time to publication was 13.0 months, and 89.7% of identified publications appeared within three years. This pattern is similar to the recent ESNR analysis, in which the median publication time was also 13.0 months and 90.4% of publications appeared within three years, and to the ESGAR 2019–2022 analysis, which also demonstrated predominantly early publication after congress presentation (10, 12). Earlier RSNA and interventional radiology studies reported that most subsequently published abstracts appeared within three years or had mean publication times of approximately 15–16 months (14, 16). These findings indicate that a three-year observation period captured most PubMed-indexed publications identified in the present cohort; however, they do not exclude later publication or publication in journals not indexed in PubMed.

An important observation is that subsequent publication did not show a monotonic increase over the four congress years. Overall publication rates ranged from 47.4% to 56.9%, and the absence of a significant year-based difference persisted when all congresses were restricted to the same 36-month follow-up period. Thus, the longer observation time available for earlier congresses does not explain the absence of a temporal trend in publication conversion. More pronounced changes were observed in the characteristics of the presented research: retrospective designs became substantially more frequent in 2021 and 2022, and national collaboration increased markedly in 2022. These findings suggest that the organization and methodology of research presented at ESCR changed more clearly during the study period than its probability of subsequent publication.

Country of origin was associated with publication outcome. Italy contributed the largest number of presentations, followed by Germany and South Korea, while Austria and the Netherlands had the highest observed publication proportions among countries with at least eight presentations. These country-level percentages require cautious interpretation because several groups were small and some had complete publication or non-publication. Geographic variation in subsequent publication has also been reported in RSNA, ECR, ESGAR, ESNR, and national radiology congress analyses (10–12, 14, 15, 17). Such differences may be influenced by multiple factors, including institutional research infrastructure, manuscript-preparation support, language, funding, and access to collaborative networks; the present study was not designed to determine the contribution of these factors.

Myocardial and coronary imaging were the most frequently represented subspecialties, consistent with the central role of cardiac MRI and cardiac CT within cardiovascular radiology. Publication outcome differed across subspecialty categories, with pulmonary imaging showing the highest observed rate. However, the pulmonary group contained only 12 presentations, so its high publication percentage should be interpreted cautiously. Variation in publication according to subspecialty or presentation topic has also been reported in previous radiology congress studies (7, 9, 10, 12, 14). The present data demonstrate heterogeneity across cardiovascular imaging topics but do not establish why particular subspecialties were more likely to reach publication.

Collaboration type was not significantly associated with publication outcome, although national and international collaborations showed numerically higher publication rates than local studies. Because these differences were not statistically significant, they should not be interpreted as evidence that multicenter collaboration increased the probability of publication in this cohort. Previous radiology congress analyses have reported associations between collaborative or multi-institutional research and publication-related outcomes (10, 12, 17). The absence of a significant association in the present study may partly reflect sample size, the affiliation-based classification strategy, in which a single foreign-affiliated co-author was sufficient for international classification, or the restriction of publication ascertainment to PubMed.

Study design was significantly associated with publication outcome. Retrospective studies had a higher publication rate than presentations classified as ‘other,’ whereas retrospective and prospective studies did not differ significantly after correction for multiple comparisons. This finding should not be interpreted as evidence that retrospective designs are methodologically superior or inherently more publishable. The ‘other’ category was heterogeneous and included reviews, systematic reviews, meta-analyses, case-based presentations, and a small number of survey-, phantom-, or technical-type original studies, some of which may not have been intended to progress to a conventional original research article. Earlier radiology studies have also reported associations between study characteristics and subsequent publication, including lower publication of technical studies in some cohorts (10, 14, 15). The observed difference in the present study may therefore partly reflect the composition of the ‘other’ category rather than study design alone.

The study period overlapped with the COVID-19 pandemic, which should be considered when interpreting the temporal changes observed. The number of oral presentations was lower in 2020 and 2021 than in 2019 and 2022, consistent with the widespread disruption of radiology research activity and scientific meetings during the early pandemic period (22, 23). The pandemic also accelerated virtual and hybrid forms of scientific communication, which may have altered congress participation and dissemination patterns (23). The higher proportion of retrospective studies in 2021 and 2022 may be compatible with changes in access to prospective recruitment and existing imaging datasets during this period, although the present data cannot establish causality. Likewise, the marked increase in national collaboration in 2022 may reflect changing research organization after the acute pandemic period rather than a stable long-term trend. The four-year study interval is therefore best interpreted as a snapshot of a dynamic period rather than evidence of a definitive secular trajectory.

This study has several limitations. First, publication status was determined exclusively through PubMed. Articles indexed only in Web of Science, Scopus, or other databases may therefore have been missed, and the observed publication rate may underestimate the overall full-text publication rate. The present study assessed whether congress presentations subsequently progressed to identifiable PubMed-indexed full-text original research articles, but did not include manuscript-submission or journal editorial histories. Therefore, non-publication cannot be attributed specifically to author non-submission, journal rejection, or other causes. Accordingly, the observed 49.8% rate should be interpreted as a publication-conversion rate rather than a journal acceptance rate, and the present data cannot determine at which stage of the post-congress publication process non-publication occurred. Second, despite independent review and consensus assessment, substantial changes in title, authorship, sample size, or study scope may have resulted in missed or uncertain matches. Third, only oral presentations were included because poster presentations were not available in the congress abstract books; the findings therefore do not represent the complete scientific output of the meetings. An additional limitation concerns potential selection bias at the congress-acceptance stage. The available dataset contained only abstracts accepted for oral presentation; rejected submissions, abstract-review scores, the number of conference reviewers assigned to each submission, and information on whether author identities were concealed during ESCR abstract selection were not available in the published congress materials. Consequently, the present study cannot determine whether author reputation, previous presentation history, or other author-level factors influenced acceptance for oral presentation. Author-level metrics such as h-index were not used to infer this bias because, without corresponding data for rejected submissions and time-matched bibliometric information, they would not permit a valid comparison. Fourth, the study-design ‘other’ category was heterogeneous, and collaboration type was determined solely from institutional affiliations; these classification strategies may have influenced the observed associations. Fifth, country-level analyses included several small groups and should be considered exploratory. The study period also overlapped with the COVID-19 pandemic, which may have influenced research activity and publication processes. Finally, although all included congresses had at least three years of follow-up by the final search date, the total follow-up duration differed between congress years; the standardized 36-month analysis was added to reduce this source of temporal imbalance. Future studies incorporating poster presentations, broader bibliographic database coverage, rejected abstract data, and extended follow-up may provide a more comprehensive assessment of research dissemination and congress-level selection in cardiovascular radiology.

## 5 Conclusion

In conclusion, approximately half of the oral presentations delivered at the ESCR congresses between 2019 and 2022 subsequently progressed to full-text original research publication indexed in PubMed, with nearly 90% of identified publications appearing within three years. Publication conversion remained relatively stable across congress years, including when a standardized 36-month follow-up period was applied, whereas the characteristics of the research presented at ESCR changed over time, particularly with respect to study design and collaboration patterns. These findings provide the first ESCR-specific benchmark for the dissemination of cardiovascular radiology research and illustrate how the structure of research presented within this rapidly evolving imaging subspecialty changed during the study period. Because the analysis was limited to accepted oral presentations and PubMed-indexed publications, it cannot assess potential author- or reviewer-related selection effects during conference acceptance and may underestimate the total rate of subsequent publication.

## Data Availability

The raw data supporting the conclusions of this article will be made available by the authors, without undue reservation.
