# Supplementary material for: Publication characteristics of oral presentations at the European Society of Cardiovascular Radiology congresses
Source: Front Cardiovasc Med. 2026 Aug 25;13:1890083. doi: 10.3389/fcvm.2026.1890083 (PMC13549968; doi:10.3389/fcvm.2026.1890083)
Supplement: Supplementary file 1 [file Table1.docx]

**Supplementary Table S1.** Publication outcomes of oral presentations according to all 32 countries of origin

| Country | Presentations (n) | Published n (%) |
| --- | --- | --- |
| Italy | 92 | 53 (57.6%) |
| Germany | 47 | 29 (61.7%) |
| South Korea | 18 | 10 (55.6%) |
| Greece | 15 | 1 (6.7%) |
| United Kingdom | 10 | 6 (60.0%) |
| Romania | 9 | 0 (0.0%) |
| Spain | 9 | 1 (11.1%) |
| Austria | 8 | 8 (100.0%) |
| France | 8 | 5 (62.5%) |
| Netherlands | 8 | 8 (100.0%) |
| Switzerland | 8 | 6 (75.0%) |
| Russia | 7 | 0 (0.0%) |
| India | 6 | 0 (0.0%) |
| Poland | 6 | 4 (66.7%) |
| United States | 6 | 6 (100.0%) |
| Belgium | 5 | 0 (0.0%) |
| Ukraine | 5 | 1 (20.0%) |
| China | 3 | 2 (66.7%) |
| Croatia | 3 | 1 (33.3%) |
| Egypt | 3 | 0 (0.0%) |
| Portugal | 2 | 0 (0.0%) |
| Sweden | 2 | 2 (100.0%) |
| Turkey | 2 | 0 (0.0%) |
| Armenia | 1 | 0 (0.0%) |
| Bulgaria | 1 | 0 (0.0%) |
| Japan | 1 | 1 (100.0%) |
| Lithuania | 1 | 0 (0.0%) |
| North Macedonia | 1 | 0 (0.0%) |
| Singapore | 1 | 0 (0.0%) |
| Slovakia | 1 | 0 (0.0%) |
| Slovenia | 1 | 0 (0.0%) |
| Taiwan | 1 | 1 (100.0%) |
